# Supplementary material for: Predicting visual function by interpreting a neuronal wiring diagram
Source: Nature. 2024 Oct 2;634(8032):113–23. doi: 10.1038/s41586-024-07953-5 (PMC11446822; doi:10.1038/s41586-024-07953-5)
Supplement: Supplementary file 6 — CRF and ERF predictions for individual TmY4 and TmY9 cells. Analogous to Supplementary Data 3, but for TmY target types. Shown are the top four monosynaptic pathways, the strongest pathway passing through each of the top ten intermediary types (ranking from Extended Data Fig. 7), and the trisynaptic pathway Tm1–TmY–Dm3–TmY (see the section entitled Prediction of spatial normalization). [file 41586_2024_7953_MOESM6_ESM.zip › DataS4/TmY9qΓèÑ/720575940608741700.pdf]

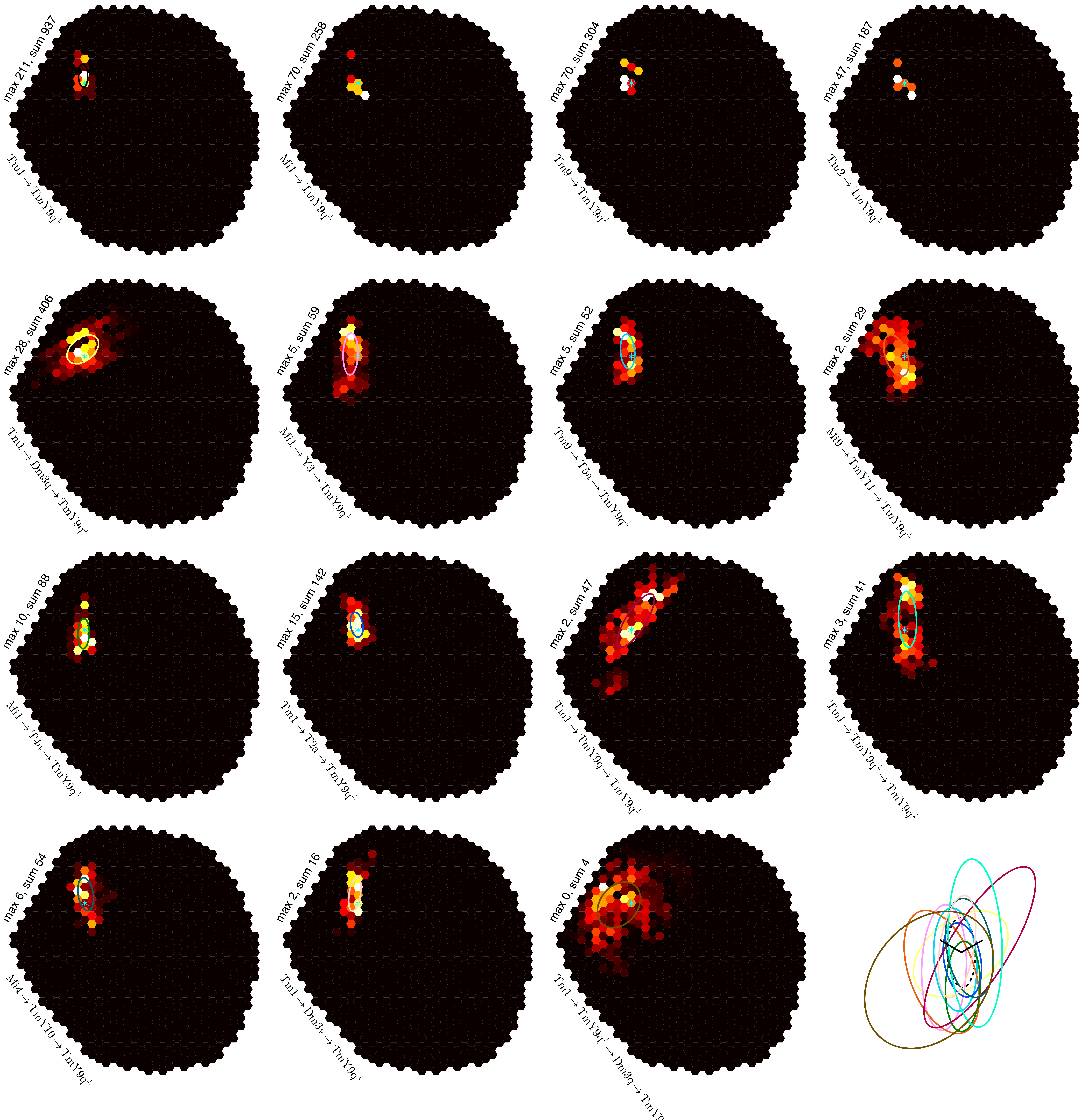
max 211, sum 937
Tm1 → TmY9q⊥
max 70, sum 258
Mi1 → TmY9q⊥
max 70, sum 304
Tm9 → TmY9q⊥
max 47, sum 187
Tm2 → TmY9q⊥
max 28, sum 406
Tm1 → Dm3q → TmY9q⊥
max 5, sum 59
Mi1 → Y3 → TmY9q⊥
max 5, sum 52
Tm9 → T5a → TmY9q⊥
max 2, sum 29
Mi9 → TmY11 → TmY9q⊥
max 10, sum 88
Mi1 → T4a → TmY9q⊥
max 15, sum 142
Tm1 → T2a → TmY9q⊥
max 2, sum 47
Tm1 → TmY9q → TmY9q⊥
max 3, sum 41
Tm1 → TmY9q⊥ → TmY9q⊥
max 6, sum 54
Mi4 → TmY10 → TmY9q⊥
max 2, sum 16
Tm1 → Dm3v → TmY9q⊥
max 0, sum 4
Tm1 → TmY9q⊥ → Dm3q → TmY9q⊥
